# Functional Assessment of Hybrid Conduits for Biomedical Applications—A Pilot Study

**DOI:** 10.3390/polym18111283

**Published:** 2026-05-23

**Authors:** Giombattista Ebanietti, Filip Borowiecki, Martina Todesco, Martina Casarin, Jacek Świniarski, Bożena Rokita, Katarzyna Kafar, Anna Karczemska, Dariusz Witkowski, Daniel Jodko, Andrea Bagno

**Affiliations:** 1 Department of Industrial Engineering, University of Padova, 35131 Padova, Italy; giombattista.ebanietti@gmail.com (G.E.); martina.todesco.2@phd.unipd.it (M.T.); 2 Institute of Turbomachinery, Lodz University of Technology, 93-005 Lodz, Poland; 242080@edu.p.lodz.pl (F.B.); 242084@edu.p.lodz.pl (K.K.); anna.karczemska@p.lodz.pl (A.K.); dariusz.witkowski@p.lodz.pl (D.W.); daniel.jodko@p.lodz.pl (D.J.); 3 Department of Surgery, Oncology and Gastroenterology, University of Padova, 35131 Padova, Italy; martina.casarin@unipd.it; 4 Department of Strength of Materials, Lodz University of Technology, 93-005 Lodz, Poland; jacek.swiniarski@p.lodz.pl; 5 Institute of Applied Radiation Chemistry, Faculty of Chemistry, Lodz University of Technology, 93-005 Lodz, Poland; bozena.rokita@p.lodz.pl

**Keywords:** hybrid materials, biomedical composite conduits, vascular surgery, urinary tract surgery, mechanical test

## Abstract

Hybrid materials, combining synthetic and biological components, leverage the biocompatibility of biological tissues—even after decellularization—alongside the mechanical strength, durability, and impermeability of synthetic polymers. This study presents the functional assessment of two hybrid conduits developed by coupling decellularized porcine pericardium and decellularized swine intestinal submucosa with a commercial polycarbonate urethane, intended for use as vascular and urinary substitutes, respectively. The response of the hybrid conduits to pulsatile flow was evaluated under physiologically relevant conditions in terms of pressure and flow rate. Their ability to withstand cyclic dilations was assessed using a dedicated image processing method that integrates classical approaches with AI-based segmentation techniques. Mechanical tests were also performed before and after hydrodynamic testing to investigate the potential effects of two different fluids—distilled water and simulated body fluid—on the hybrid materials following cyclic hydrodynamic stimulation. The results demonstrate that hybrid conduits deserve to be subjected to further evaluations to prove their potential use as substitutes in vascular and urological surgical applications.

## 1. Introduction

Atherosclerosis is one of the most common cardiovascular diseases and represents the leading cause of myocardial infarction and stroke—when affecting the coronary and cerebral arteries—and limb ischemia—when involving peripheral arteries [1]. It is a slow-progressing degenerative disease characterized by the accumulation of lipids, inflammatory cells, smooth muscle cells, and necrotic cellular debris within the intimal layer of the blood vessel wall [2]. This process gradually impairs blood flow, reducing it by over 50% within the vascular lumen and, in severe cases, may result in complete vascular occlusion [3]. When feasible, replacement of the damaged vessel with an autologous one is the preferred approach: the saphenous vein and internal thoracic artery represent the gold standard grafts for small-diameter vessel replacement. Despite their excellent patency, these grafts require invasive harvesting procedures, are often unsuitable, and still exhibit failure rates of approximately 50% within a decade [4,5]. Alternatively, synthetic vascular grafts—such as those made of expanded polytetrafluoroethylene or polyethylene terephthalate—offer good performance in terms of impermeability, flexibility, and compliance, but are associated with long-term risks of infection, inflammation, thrombogenicity, and intimal hyperplasia [6]. Synthetic grafts, particularly for small-diameter vessel replacement, require sustained anticoagulant therapy, which interferes with the physiological hemostatic process. This is especially critical when synthetic grafts are used for vascular access in hemodialysis patients with kidney failure [7].

In the urological field, bladder cancer represents a condition of foremost clinical interest. It is an epithelial neoplasm that primarily arises from the urothelium and ranks as the ninth most common cancer worldwide, with a markedly higher prevalence in males (male-to-female ratio up to 4:1) and a peak incidence during the sixth to seventh decade of life [8,9]. Radical cystectomy is the gold standard treatment for patients with muscle-invasive bladder cancer and for those with high risk of non-muscle-invasive bladder cancer, who are unresponsive to intravesical therapy or present unfavorable features for transurethral resection [10]. The procedure involves the removal of the bladder—along with the prostate in men, or the uterus, part of the vagina, and urethra in women—extended bilateral pelvic lymph node dissection, and construction of a urinary diversion that redirects the urine away from the bladder. To restore urinary functionality, an autologous intestinal segment is typically used to create the diversion. However, bowel tract removal is associated with several complications that significantly impact patients’ quality of life [11].

Vascular and urological prostheses represent fundamental solutions in modern surgery for restoring and replacing arteries, veins, and urinary tracts [12,13,14,15]. The demand for biocompatible materials to develop vascular and urological substitutes remains a key challenge in biomaterials science. Autologous tissues, which are ideal in terms of biocompatibility, are limited by reduced availability and, at times, poor quality due to underlying medical conditions—for instance, atherosclerosis induces structural changes throughout the entire vasculature. These constraints drive the need to develop alternative materials capable of replicating the functionality of native tissues.

Hybrid materials, merging biological and synthetic components, offer a promising solution [16]. By combining the regenerative potential of biological tissues with the mechanical strength, impermeability, and durability of synthetic polymers, hybrid materials can achieve properties that neither component could attain individually.

Decellularized porcine pericardium (DPP), valued for its fibrous collagen matrix, provides a biocompatible scaffold widely used in bioprosthetic heart valves and vascular grafts. However, DPP’s limited mechanical strength and inherent permeability need to be addressed through the incorporation of synthetic polymers. In a recent study, DPP was coated with Chronoflex AR (CF AR), a polycarbonate urethane recognized for its biocompatibility, mechanical properties, and resistance to biodegradation [17]. The hybrid material was fabricated and shaped into a tubular structure suitable for use as a vascular graft. Mechanical tests were performed to assess the hybrid conduit’s mechanical properties, comparing them to those of DPP alone and the polymer alone.

The pericardium-based hybrid material was further evaluated through preliminary in vivo studies in rats to assess its biocompatibility. Compared to the polymer alone, it exhibited a higher degree of integration with the host tissue, increased neovascularization around the implant, a reduced fibrotic reaction, and lower levels of inflammation [18].

Small intestinal submucosa (SIS) is considered a promising scaffold due to several remarkable properties: biocompatibility, low immunogenicity, high biological activity, and biodegradability. SIS is rich in collagen, proteoglycans, glycoproteins, and growth factors, making it a suitable candidate for tissue engineering applications and clinical use [19]. Furthermore, the FDA has approved the use of SIS in humans for several urogenital procedures, including hernia repair [20], cystoplasties [21], ureteral reconstructions [22], stress incontinence [23], Peyronie’s disease [24], penile chordee [25], and urethral reconstruction for hypospadias and strictures [26]. These findings support the potential use of decellularized SIS for urinary conduit construction, eliminating the need to harvest healthy autologous intestinal segments currently employed in urinary diversion procedures [27]. Following the same strategy applied to pericardial tissue, decellularized SIS is coupled with CF AR to enhance its mechanical performance and provide an impermeable barrier.

Our previous investigations have already shown some advantages of the proposed hybrid material approach for biomedical applications. This work aims to perform a preliminary evaluation of hybrid conduits to assess their mechanical response under load before and after exposure to different water-based environments. Hybrid conduits were exposed to a pulsatile flow—with distilled water (DW) or simulated body fluid (SBF)—under para-physiological conditions in terms of flow rates and pressures. Their compliance was measured throughout the testing phase and their mechanical properties were compared before and after functional testing. The results obtained in this preliminary study support the hypothesis that hybrid conduits hold potential for application in graft manufacturing. However, a more comprehensive chemical, mechanical, and functional analysis, followed by biocompatibility assessment, is required to achieve statistical significance and to meet the specific requirements of vascular and urological substitutes.

## 2. Materials and Methods

This section explains how the investigated hybrid conduits were manufactured and describes all the protocols used to analyze them.

### 2.1. Hybrid Conduits Fabrication

Hybrid conduits (HCs) were obtained by coupling decellularized porcine pericardium and decellularized swine intestinal submucosa (SIS) with Chronoflex AR (AdvanSource Biomaterials, Wilmington, MA, USA). Chronoflex AR is a commercial polycarbonate urethane, supplied as 22% (*w*/*v*) solutions in N,N-dimethylacetamide (DMAc), recognized for its bio- and hemocompatibility.

Fresh pericardia and small intestines were harvested from healthy Duroc pigs (9–14 months old and weighing between 140 and 170 kg) at local slaughterhouses and processed within three hours post-mortem. All slaughterhouse procedures were conducted in compliance with European Regulation EC 1099/2009 on animal health and welfare, under the supervision of the Italian government and approved by the associated legal authorities on animal welfare (Food and Consumer Product Safety Authority). Tissue samples were treated and decellularized as previously described [19,28,29]. Briefly, pericardial tissues were preliminarily protected from lytic degradation by protease inhibitor solution, followed by alternating hypo/hypertonic solutions with Tergitol (1–0.1% *v*/*v*) and 10 mM sodium cholate to extract cellular components. Extractions were carried out in a solution containing 10 mM sodium ascorbate and 5 mM ethylenediaminetetraacetic acid (EDTA) under nitrogen atmosphere to prevent oxidation. After washing with saline solution, 10% isopropanol was used. Finally, tissues were exposed to a nonspecific endonuclease (Benzonase) in equilibration buffer (50 mM Tris-HCl, 1 mM MgCl2) at 37 °C for 48 h to fragment double- and single-stranded nucleic acids [27]. All reagents were supplied by Sigma-Aldrich (Saint Louis, MO, USA).

SIS was decellularized with an optimized protocol involving mechanical agitation and protease inhibitors, alternated with hypotonic and hypertonic solutions. Native tissues were then incubated with Tergitol and sodium cholate, with intermediate washing cycles in PBS or saline solution. Then, an alcohol-based solution was used for tissue decontamination and delipidation. Finally, residual DNA fragments were removed by Benzonase [19].

**Figure 1:**
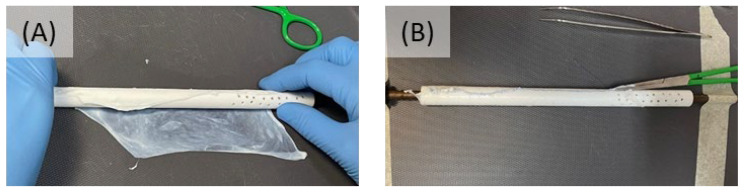
(**A**) Decellularized DPP wrapped around the 13 mm diameter PTFE mandrel, with the serous membrane facing inward to ensure the correct functional orientation of the conduit. (**B**) Decellularized pericardium sutured along the junction line with a 5-0 Prolene thread.

**Figure 2:**
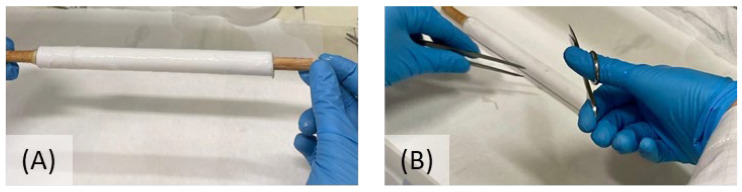
(**A**) Decellularized SIS placed onto the PTFE mandrel (16 mm diameter). (**B**) Removal of excess filaments from the outer surface of the SIS to obtain a smooth and uniform surface, ensuring optimal adhesion during coating.

Pericardium-based HCs were produced by wrapping the decellularized tissue over a Teflon mandrel (13 mm diameter), carefully suturing its edges with a 5-gauge Prolene thread (Ethicon Inc., Raritan, NJ, USA), and drying with Whatman filter paper (Sigma-Aldrich, Burlington, MA, USA). After removing air bubbles under vacuum, the degassed polymer solution was deposited onto the outer surface of the pericardium while the mandrel was kept rotating (Figure 1). The construct was then placed in a vacuum oven (Raypa Vacuum Drying Oven EV-50, RAYPA R. Espinar S.L., Barcelona, Spain) for complete drying at 38 °C for 24 h. Finally, each HC was carefully separated from the Teflon mandrel. Two conduits were prepared—DPP-1 and DPP-2—one designated for functional testing with DW and one for testing with SBF. HCs based on decellularized SIS were similarly created. Decellularized SIS was applied around a Teflon mandrel (16 mm diameter), and excess filaments on the outer surface were carefully removed (Figure 2). To promote optimal adhesion of the polymer and obtain a smooth and uniform surface, SIS was dried using Whatman filter paper. Then, the mandrel with SIS was kept rotating, and the degassed polymer solution was deposited onto the outer surface, then placed into the vacuum oven under a chemical fume hood for solvent removal (24 h at 38 °C). Finally, each HC was carefully removed from the mandrel. Two HCs were prepared (SIS-1 and SIS-2), one for the functional tests with DW and one for the test with SBF.

### 2.2. Functional Assessment

**Figure 3:**
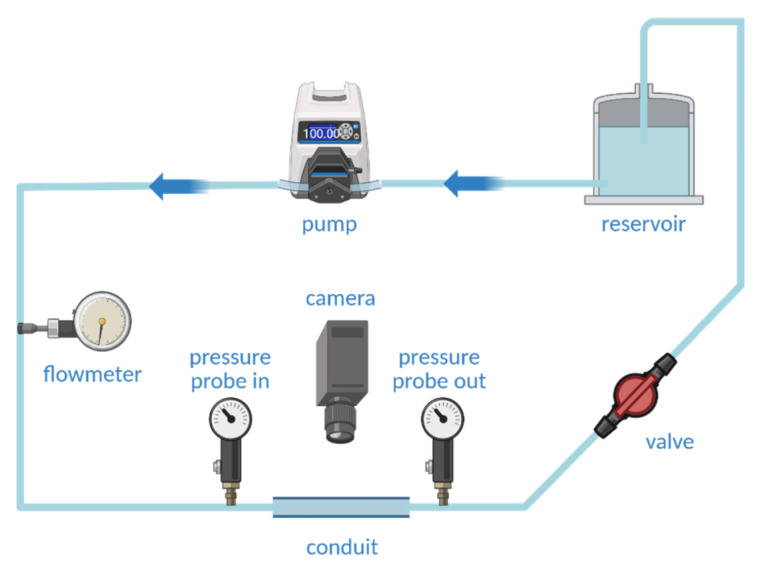
Schematic of the hydraulic circuit for the functional assessment of the hybrid conduits: the fluid (DW or SBF) is pumped by a peristaltic pump through the circuit in which the conduit is located; two pressure probes are inserted upstream and downstream of the conduit; pressure is manually controlled by operating the mechanical valve; flow rate is measured by the flow meter; a high-speed camera enables real-time acquisition of conduit dilation. Created in BioRender. Bagno, A. (2026) https://BioRender.com/etmatqa (accessed on 15 May 2026).

For the functional assessment of the hybrid conduits, a closed hydraulic circuit was designed and assembled (Figure 3). It included the following components: the head of a peristaltic pump (TH25, Aqua-Trend, Łódź, Poland) driven by a BM56M servo motor (24 V DC, 50 W) controlled via the Ezi-SERVO-PR driver; a HighSpeedStar high-speed camera (LaVision GmbH, Göttingen, Germany) positioned over the conduit, enabling image acquisition of its dynamic behavior; an ultrasonic flowmeter SONOFLOW CO.55/100 V2.0 (SONOTEC, Halle, Germany) with the frequency output 0–20 kHz and the accuracy ± 20 mL/min for the range 0–1000 mL/min; two tiny pressure probes ABPDANT005PGAA5 (Honeywell, Charlotte, NC, USA) with the 1.5% of total error band and the accuracy ± 0.25% of full scale span (FSS = difference between the output signal measured at the maximum (Pmax) and minimum (Pmin) limits of the pressure range; output update rate ~1 kHz). Flexible plastic tubes were used to connect all components. A rigid tube (11 mm inner and 16 mm outer diameter, 30 cm length) was inserted upstream to stabilize the flow before entering the conduit under investigation. The conduit was then mounted using two T-junctions—one at the inlet and one at the outlet—while the remaining ports of the junctions were connected to rigid tubes mounted on supports.

Signal acquisition from both pressure probes and flow meter was carried out using NI USB-6363, X Series DAQ (National Instruments, Warsaw, Poland), connected via SCB-68A Noise Rejecting, Shielded I/O Connector Block.

The image acquisition system was based on DaVis 8.4.0 software (LaVision GmbH, Göttingen, Germany). To enhance image quality, a black paper sheet was placed beneath the conduit and illumination was arranged uniformly to reduce reflections. Images were acquired at 60 Hz, for a total of 100 frames per sequence, corresponding to an overall duration of 1666.6 ms. Depending on experimental requirements, frames were exported either in grayscale or as binary images to provide sufficient contrast for segmentation while optimizing memory allocation. This setup allowed precise monitoring of the conduit deformation during pulsatile flow tests and ensured that the acquired data were reliably processed for subsequent analysis.

Two different fluids were used for the functional assessment: distilled water (DW) and simulated body fluid (SBF). The latter was selected to evaluate the possible effects of salt deposition onto the inner surface of the conduit.

SBF was prepared according to the formulation proposed by Oyane et al. [30], which reproduces the ion concentrations of human blood plasma (Table A1), and was freshly prepared immediately before each experiment.

Experiments were conducted at different flow rates, ranging from 220 mL/min to 1000 mL/min (220, 370, 500, 750, and 1000 mL/min), representative of those expected in the human cardiovascular system [31,32]. For each flow rate value, pressure was increased from the baseline up to the systolic blood pressure of 16 kPa (120 mmHg) in increments of 1 kPa. Changes in conduit diameter induced by the pulsatile flow generated by the two-roller peristaltic pump were monitored using the video acquisition system, while upstream and downstream pressure and flow rate values were recorded simultaneously. Pressure increase was achieved by partially closing the mechanical valve positioned downstream of the conduit.

### 2.3. Image-Based Processing for HC Deformation Analysis

To analyze the behavior of HCs under pulsatile flow at different flow rate values, acquired images were processed using a combination of classical methods and AI-based segmentation techniques. Grayscale images were pre-processed to reduce noise and enhance contrast; in some cases, binary transformations and morphological operations were applied to obtain continuous masks of the conduit lumen. In parallel, a deep learning model based on U-Net++ with a ResNet-50 encoder was developed and trained on a dedicated dataset of images and masks, enabling automatic segmentation of each conduit.

Geometric parameters, such as minimum and maximum diameters (D({min}) and D({max}), respectively), were extracted from the segmentation results. Conduit diameter values were then synchronized with the pressure signals, allowing each frame to be associated with the corresponding instantaneous diameter and pressure value. This approach provided both qualitative visualizations (e.g., images and GIFs) and quantitative metrics (e.g., maximum, minimum, and mean diameters, and Dmax/Dmin ratio).

#### 2.3.1. Image Processing

Images acquired during each functional test were processed in grayscale to preserve the intensity information necessary for accurate identification of the conduit lumen. When the grayscale processing did not allow a clear definition of conduit contours, binary images were used to enhance the contrast between conduit and background to facilitate segmentation.

Two distinct codes were employed for image processing, specifically developed for each of the two different types of images. In both cases, image processing included morphological operations, such as opening and closing, to reduce noise and regularize contours, followed by a flood-fill procedure to fill any internal cavities, thus obtaining a continuous mask of the conduit lumen. Diameter measurement was performed in two separate steps. In the first step, the conduit diameter was measured for each frame along all columns of the region of interest (ROI): the column with the highest standard deviation across frames was defined as the “variable” diameter, representing the position characterized by the largest deformation. In the second step, diameters were measured on all frames, also recording the “fixed” diameters at predefined positions (300, 600, 900 px).

#### 2.3.2. Deep Learning Model for Conduit Segmentation

When the aforementioned image processing method did not yield well-defined conduit contours, a supervised learning approach based on convolutional neural networks with an encoder–decoder architecture was adopted. In particular, variants of U-Net and U-Net++ were employed, as their architectures are effective for biomedical image segmentation.

The encoder was used to extract increasingly complex features from the input image, while the decoder reconstructed the segmented mask by combining these representations with spatial details through skip connections. Three models were tested: the first two employed ResNet-18 and ResNet-34 encoders, both trained with a combined binary cross-entropy (BCE) and Dice loss function, simultaneously accounting for the discrepancy between predictions and binary masks and the overlap between predicted and ground truth masks, proving particularly effective for small target regions.

The final adopted model was a U-Net++ with a pretrained ResNet-50 encoder, characterized by nested skip connections—that is, multi-level skip connections that reduce the semantic gap between low- and high-level features and enhance the ability to segment fine and complex structures. The model was designed to process single-channel grayscale images and output binary masks (0 and 1), clearly distinguishing the foreground (conduit) from the background.

A total of approximately 3300 images were used, organized as image–mask pairs with a unique naming convention based on the concatenation of the test folder name and the original filename. This structure ensured a direct and unambiguous correspondence between images and masks.

Although the dataset originated from a single conduit type (SIS under SBF conditions), it was acquired across multiple experimental campaigns characterized by varying flow rates (370–1000 mL/min) and pressure levels (8–16 kPa), thereby introducing a significant degree of intra-condition variability in the deformation states of the conduit.

The dataset was divided as follows: 70% for training, 15% for validation, and 15% for testing, following the standard supervised learning scheme to ensure proper model generalization. To further improve robustness and reduce the risk of overfitting, extensive data augmentation was applied using the Albumentations library, including resizing to 256 × 256 pixels, random rotations up to 180°, brightness, contrast and gamma variations, histogram equalization, pixel normalization, and conversion into PyTorch tensors (PyThorch 2.1).

It should be noted that the dataset split was performed at the frame level, which may introduce a degree of correlation between training, validation, and test samples due to the temporal continuity of the acquired sequences, as consecutive frames may belong to the same experimental run.

Training was carried out using the Adam optimizer (learning rate = 1 × 10^−3^, weight decay = 1 × 10^−4^), combined with a ReduceLROnPlateau scheduler—which automatically decreases the learning rate when the validation loss plateaus—and early stopping (patience = 7 epochs), which halts training in the absence of improvements, thereby reducing computational time and preventing overfitting. The evaluation metric adopted was the Dice coefficient, which is particularly suited for assessing segmentation quality. During training, the best-performing model was automatically saved. Additionally, a prediction function was implemented to enable the generation of binary masks from new images with automatic resizing to their original resolution.

All training and validation operations were performed on Google Colab using the NVIDIA T4 GPU runtime, which significantly reduced computational time and allowed effective dataset management.

**Figure 4:**
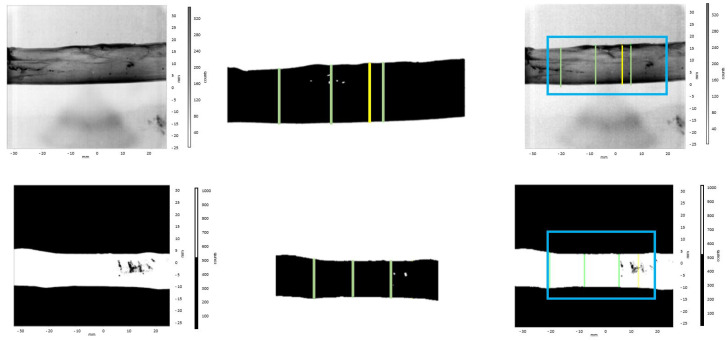
Sequence of processing steps for the analysis of images acquired during functional tests. First row (grayscale image): the original image (**left**), the segmented mask (**center**), and the final result (**right**), in which the ROI is highlighted in blue, the fixed diameters in green, and the ‘variable’ diameter in yellow. Second row (binary image): the original image (**left**), the segmented mask (**center**), and the final result (**right**), in which the ROI is highlighted in blue, the fixed diameters in green, and the ‘variable’ diameter in yellow.

The U-Net++ segmentation model with a ResNet-50 encoder was integrated into the image processing pipeline using the segmentation_models_pytorch library. After loading the trained model on Google Colab, it was set to evaluation mode on an NVIDIA T4 GPU, enabling real-time processing of input images. Each grayscale image underwent a pre-processing procedure defined with Albumentations, including resizing, normalization, and conversion into a PyTorch tensor. The predict_mask function managed model inference, generating a binary segmentation mask that was subsequently rescaled to the original image resolution. This integration enabled the direct inclusion of automated segmentation within the processing workflow, which was essential for isolating the conduits. The generated masks were used to extract relevant geometric parameters—minimum, maximum, and mean diameter values—calculated for each selected pixel column. Numerical results were stored in .csv files, while segmentation outcomes were documented through processed images and animated GIFs displaying measurement lines on the original images alongside the corresponding segmented masks (Figure 4). This approach enabled combined quantitative analysis and visual inspection of the HCs, integrating AI-based segmentation with geometric post-processing within a unified framework.

To further assess the robustness and generalization capability of the trained model, it was subsequently applied to independent datasets not included in the training phase, acquired from different conduit types under experimental conditions not encountered during training. Specifically, the model was tested on a DPP-1 conduit in distilled water (DW) and on an SIS-2 conduit also under DW conditions. In both cases, the model was used exclusively in inference mode for automated segmentation and subsequent quantitative analysis of conduit diameter dynamics. These additional results provide qualitative evidence of the model’s ability to generalize across different experimental domains, although they were not intended as a complete external validation.

#### 2.3.3. Integration of the Deep Learning Model into Image Processing

To perform an integrated analysis of conduit deformation as a function of pressure, images acquired during functional tests were synchronized with pressure data, and diameters were measured from the images using Python 3.12. Conduit diameters were extracted from each frame—processed either through image filtering or using the U-Net++ model—and subsequently converted from pixels to millimeters using a calibration scaling factor. Further details are reported in Appendix A.

### 2.4. Mechanical Tests

Mechanical tests were performed using the Zwick Roell Z005 (ZwickRoell Sp. z o.o. Sp. k., Wroclaw, Poland) universal testing machine (ProLine series), capable of applying loads up to 5 kN and equipped with an AC servo motor drive system with holding brake, ensuring high precision in crosshead movements (maximum deformation: 1000 mm, displacement resolution: 0.0348 µm) and an adjustable speed ranging from 0.0005 to 1500 mm/min. Force measurement complies with DIN EN ISO 7500-1 and ASTM E4 standards, with an accuracy class of 0.5/1 depending on the load cell used, covering an extended measurement range up to 165% of Fmax.

Tensile tests were performed on DPP-2 samples, while ring tests were performed on SIS-1 and SIS-2 samples. The experiments were managed through the integrated software testXpert III, which enabled data acquisition and preliminary processing. Subsequently, raw data were imported into MATLAB R2023b for analysis, from which stress–strain curves were drawn and relevant mechanical parameters were calculated. Specifically, engineering stress (σ, Pa) was defined as the applied force divided by the initial cross-sectional area of the sample:

$$\sigma =\frac{F}{A}$$

and the strain (ε, %) as the ratio between the deformation achieved by the sample and the gauge length:

$$\varepsilon =\frac{L-L_{0}}{L_{0}}$$

From the stress–strain curve of each sample, the following parameters were calculated: Ultimate Tensile Strength (UTS) and Failure Strain (FS), corresponding to the maximum stress and the maximum strain at failure, respectively.

#### 2.4.1. Ring Tensile Test on SIS-1 and SIS-2

Decellularized SIS samples were subjected to ring tensile tests to failure to evaluate their mechanical behavior. Four specimens, each 3 mm in width, were cut from the conduits. Samples were analyzed under three different conditions: before the hydrodynamic test following simple immersion in distilled water (DW); after the hydrodynamic test in the specific testing fluid, with SIS-1 tested in SBF and SIS-2 in DW. SIS-1 samples were additionally kept immersed in SBF for 15 days following the hydrodynamic test to assess the potential effects of calcification.

Mechanical tests were conducted by mounting the samples on T-grip fixtures with an initial gauge length (L_0_) of 24 mm, applying a constant strain rate of 20 mm/min until failure.

From the stress–strain curves, two elastic moduli were calculated as the slope of the curve within the linear regions at the beginning and at the end of the curve, fitted using the least squares method. The strain interval considered for the evaluation of the initial modulus E_1_ was 5–10% for all samples. For the final modulus E_2_, the range was 350–375%, except for samples that failed at lower strains, for which a range of 240–257% was considered instead.

#### 2.4.2. Tensile Test on DPP-1 and DPP-2

Initially, both DPP-1 and DPP-2 samples were intended for uniaxial tensile tests to failure to evaluate their mechanical properties. Four specimens, 3 mm in length and 0.8 mm in thickness, were prepared; however, DPP-1 samples were excluded from mechanical characterization—both before and after the hydrodynamic test in DW—due to significant leakage caused by imperfect sutures.

DPP-2 samples were assessed under three conditions: before the hydrodynamic test following simple immersion in DW; after the hydrodynamic test in SBF; and after an additional 15-day immersion in SBF following the test, to evaluate the potential effects of calcification.

Mechanical tests were performed by mounting the specimens in standard tensile test grips with an initial gauge length (L_0_) of 24 mm, applying a constant strain rate of 20 mm/min until failure. For DPP-2 samples, the elastic modulus was calculated within the 2–8% linear deformation region.

## 3. Results

This section is divided into four subsections describing the results obtained from preliminary tests of the test stand, hydrodynamic functional experiments, advanced analysis of wall deformation during the functional tests, and tensile tests.

### 3.1. Experimental Apparatus Set-Up

The experimental apparatus was preliminarily tested to assess its performance in terms of flow rate stability and pressure control. This step ensured that the hydrodynamic conditions imposed on the conduits were reproducible and consistent with the target nominal values. The raw data acquired by the monitoring system were processed for each nominal operating condition, extracting the minimum, mean, and maximum values of both flow rate and inlet pressure. This preliminary analysis provided a quantitative characterization of the conditions imposed by the experimental apparatus, which serves as the reference framework for interpreting the results presented in the following sections.

### 3.2. Image Processing Results

A deep learning approach based on a U-Net++ architecture with a ResNet-50 encoder was employed to enhance image processing quality when necessary. The model was trained on a dataset of 3300 images consisting of image–mask pairs, split into 2310 training images (70%), 495 validation images (15%), and 495 test images (15%). A batch size of 8 images was used during training, balancing computational efficiency with training stability. To improve model robustness and mitigate the risk of overfitting, a data augmentation pipeline was implemented using the Albumentations library. All 2310 training images were subjected to random transformations, with the following specifications:

- resizing to 256 × 256 pixels to standardize the input dimensions;
- random rotations of up to 180°, with a probability of 0.7;
- random brightness and contrast variations within ±30%, with a probability of 0.7;
- random gamma adjustment between 0.8 and 1.2, with a probability of 0.5;
- histogram equalization, applied with a probability of 0.3;
- pixel normalization with a mean of 0.5 and a standard deviation of 0.5;
- final conversion to PyTorch tensors.

These transformations generated a diverse set of augmented images, enhancing the model’s ability to generalize across varying experimental conditions, including differences in brightness, contrast, and orientation.

**Figure A1:**
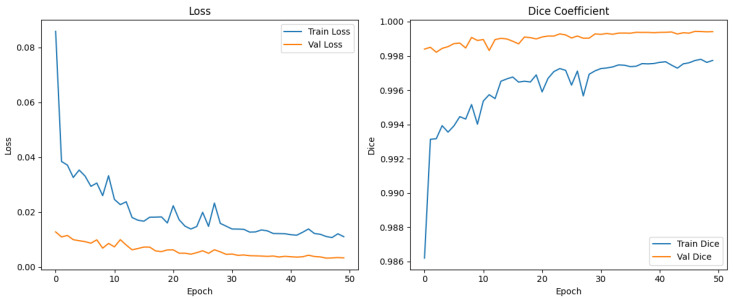
Loss function and Dice coefficient trends monitored over 50 training epochs.

The training process was monitored using the loss function, the Dice coefficient, and the Intersection over Union (IoU). The Dice coefficient measures the overlap between the predicted mask and the ground truth mask, ranging from 0 to 1, where 1 indicates perfect agreement. The IoU quantifies the ratio between the intersection and the union of the predicted and ground truth areas, providing a stricter assessment of segmentation accuracy. During training, the loss progressively decreased, while the Dice coefficient and IoU increased toward values approaching 1. At the end of training (epoch 50), the validation loss was 0.0033, with a mean Dice coefficient of 0.9994 and an IoU of 0.9988 (Figure A1).

The trained model was evaluated on 495 independent images using several performance metrics:

- Precision: the proportion of pixels predicted as positive that are correctly classified, reflecting the model’s ability to minimize false positives;
- Recall (or Sensitivity): the model’s ability to correctly identify all pixels belonging to the conduit, capturing the impact of false negatives;
- F1-score: the harmonic mean of Precision and Recall, providing a balanced measure of the model’s ability to minimize both false positives and false negatives.

**Figure A2:**
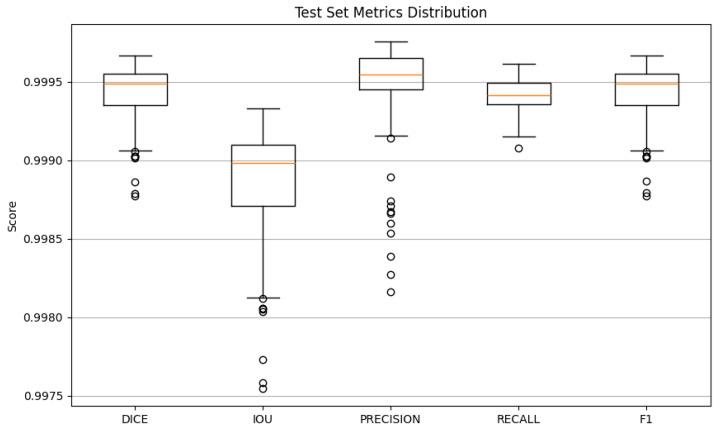
Boxplots of segmentation metrics evaluated on the test set: distribution of Dice coefficient, Intersection over Union (IoU), Precision, Recall, and F1-score across 495 test images.

The results obtained were excellent, with Dice, IoU, Precision, Recall, and F1-score all exceeding 0.998. The metric distributions were highly compact, as shown in the boxplots in Figure A2, indicating minimal variability across samples.

A qualitative analysis was conducted by visually comparing the predicted masks with the ground truth. To further assess spatial accuracy, pixel-wise error maps were generated accordingly. Errors were minimal and mostly localized along the conduit edges, consistent with typical annotation uncertainties.

### 3.3. Results of Functional Tests

Functional investigations on hybrid conduits were conducted over a flow rate range of 220 to 1000 mL/min and at increasing pressures up to 16 kPa (120 mmHg). For biologically relevant evaluation, the main focus was placed on the physiological range, corresponding to 370–500 mL/min and 8–11 kPa (60–83 mmHg). These values are representative of the typical hemodynamic conditions found in medium-sized arteries, such as the femoral (350 mL/min) and iliac (371–766 mL/min) arteries.

As previously described, diameter changes were measured using two approaches: (1) a classical image processing pipeline applied to grayscale or binary images; and (2) a convolutional neural network-based segmentation. In both methods, conduit diameter changes were measured at fixed positions (300, 600, and 900 pixels) along each conduit to monitor localized deformations, and at the column exhibiting the highest standard deviation (variable position), identifying the region of greatest dynamic dilation.

For each hybrid conduit, the results are presented as heatmaps showing the Dmax/Dmin ratio as a function of flow rate and pressure. This ratio normalizes the luminal excursion, providing a relative measure of conduit compliance and enabling the identification of conditions that deviate most from physiological behavior or closely approximate the hemodynamic values typical of natural arteries.

#### 3.3.1. Results for the SIS-1 Conduit

**Figure 5:**
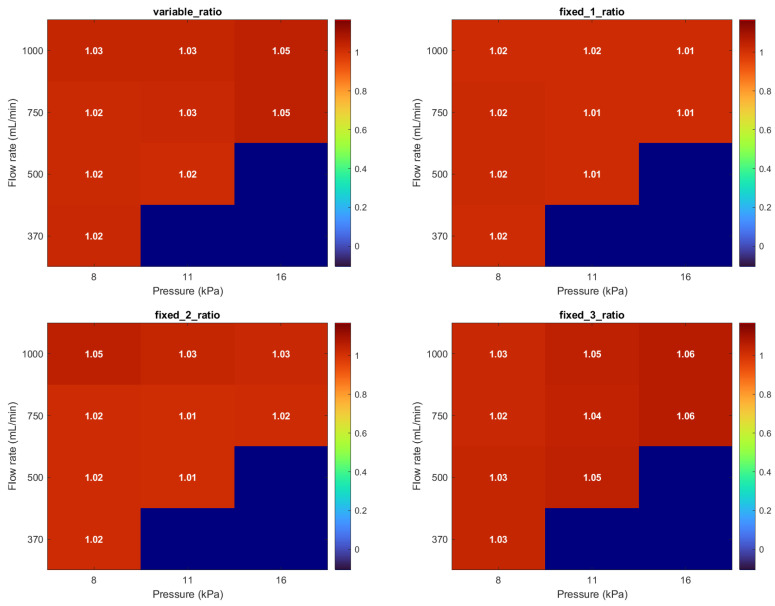
Conduit SIS-1 tested in SBF: heatmaps of the Dmax/Dmin ratio as a function of flow rate and pressure. Blue areas indicate conditions that were not experimentally investigated.

The SIS-1 conduit was tested with SBF. The quantitative assessment of diameter changes, both at fixed positions and along the variable column, is summarized in terms of the Dmax/Dmin ratio as a function of flow rate and pressure (Figure 5). The results indicate stable behavior across the entire operating range, with minimal diameter changes (i.e., ratio values close to 1). The highest compliance variation was observed under the most demanding hemodynamic conditions, where the maximum Dmax/Dmin ratio reached 1.06 at 16 kPa and elevated flow rates (750 and 1000 mL/min). Conversely, within the physiological range (8–11 kPa, 370–500 mL/min), the conduit exhibited Dmax/Dmin values consistently between 1.01 and 1.02.

#### 3.3.2. Results for the SIS-2 Conduit

**Figure 6:**
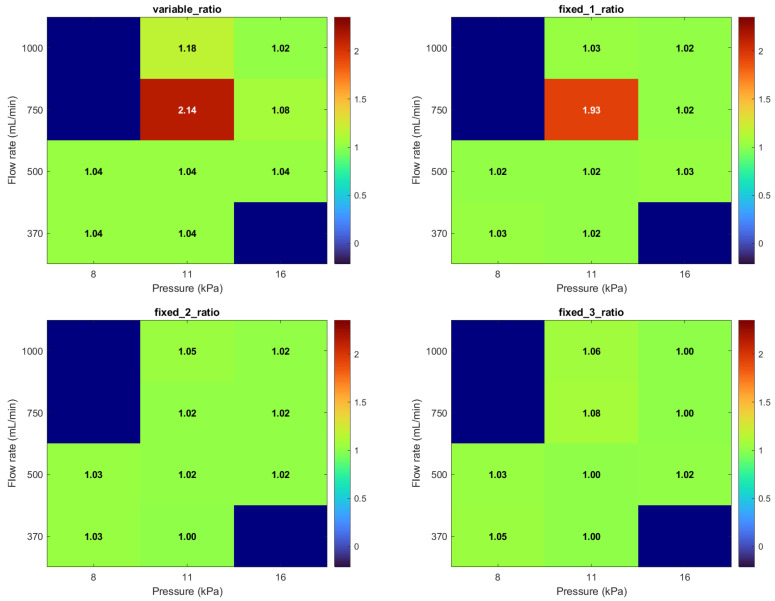
Conduit SIS-2 tested in distilled water: heatmaps of the Dmax/Dmin ratio as a function of flow rate and pressure. The anomalous value measured at 11 kPa and 750 mL/min may be attributed to discontinuities in the conduit wall segmentation. Blue areas indicate conditions that were not experimentally investigated.

Conduit SIS-2 was tested in distilled water, and the acquired images were processed using the U-Net++ convolutional neural network model with a ResNet-50 encoder, which was essential for achieving accurate contour segmentation. This analysis yielded reliable results, with the only exception being an error detected during the test at 750 mL/min and 11 kPa. Under this condition, the presence of a central region containing numerous black pixels prevented the proper identification of the conduit profile. Furthermore, the extremities lacked complete segmentation continuity, likely because the model was trained to recognize the conduit itself rather than the junction areas. Nevertheless, this limitation did not affect the measurements, as both the fixed diameter positions and the region of interest were defined within the central portion of the conduit, thereby excluding the extremities from the analysis. The Dmax/Dmin ratio values ranged between 1.00 and 2.14, with the maximum value observed at 750 mL/min and 11 kPa (Figure 6).

#### 3.3.3. Results for the DPP-1 Conduit

**Figure 7:**
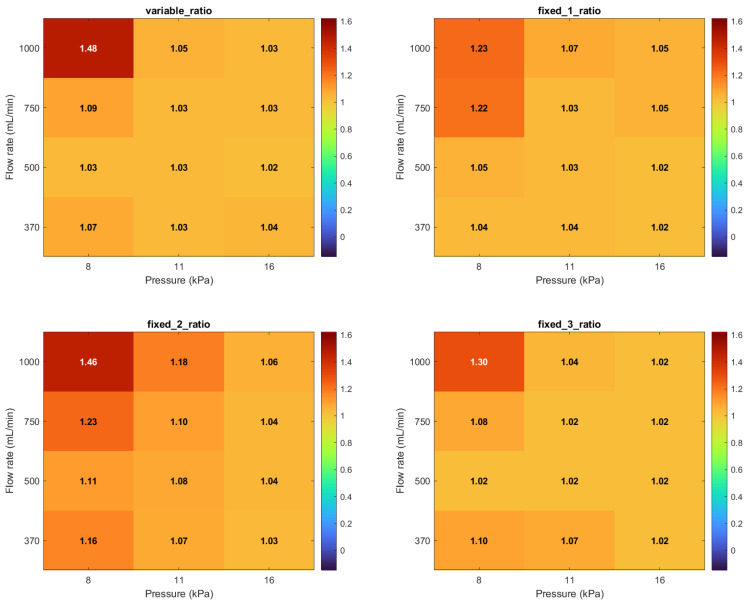
Conduit DPP-1 tested in distilled water: heatmaps of the Dmax/Dmin ratio as a function of flow rate and pressure.

Conduit DPP-1 was tested in distilled water: grayscale images were segmented using a U-Net++ convolutional neural network with a ResNet-50 encoder, which ensured accurate delineation of the conduit contours. During the test at 370 mL/min and 8 kPa, discontinuities along the conduit wall were observed, attributed to imperfect sutures. To allow testing to continue, an instant adhesive (Kropelka, Fenedur S.A., Montevideo, Uruguay) was applied, ensuring sample integrity without affecting the measurements. Nevertheless, the adhesive was applied locally (at 4 individual points along the suture line), without altering the wall behavior observed before the occurrence of leakage for the same pressure excitation. The Dmax/Dmin ratio ranged between 1.06 and 1.48, with the maximum value observed at 1000 mL/min and 8 kPa, where all diameter measurements reached notable peaks: 1.48 for the variable diameter, 1.23 for diameter 1, 1.46 for diameter 2, and 1.30 for diameter 3 (Figure 7). Within the physiological reference range, the conduit displayed consistent and regular values, with a maximum of 1.16 recorded at 370 mL/min and 8 kPa.

#### 3.3.4. Results for the DPP-2 Conduit

**Figure 8:**
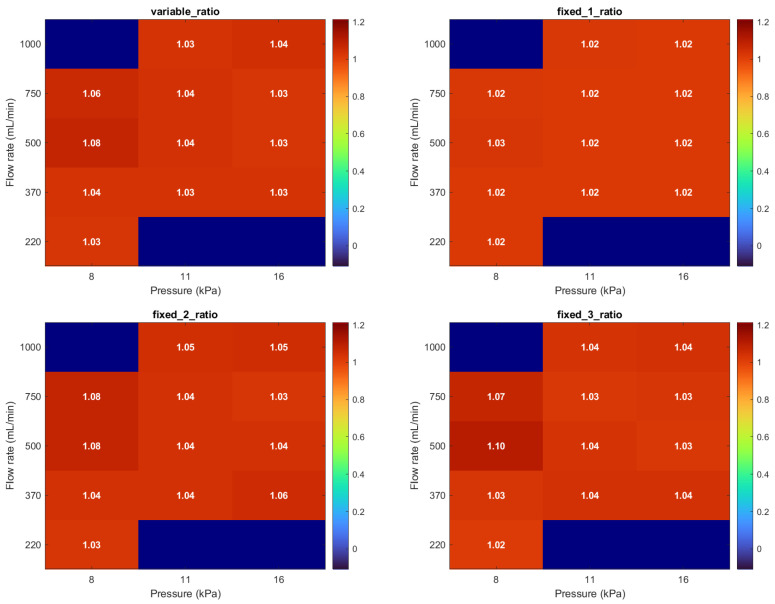
Conduit DPP-2 tested in SBF: heatmaps of the Dmax/Dmin ratio as a function of flow rate and pressure. Blue areas indicate conditions that were not experimentally investigated.

Conduit DPP-2 was tested in SBF: it maintained structural integrity throughout the entire experiment, with no evidence of leakage or damage. The acquired images enabled clear and error-free segmentation, ensuring a reliable analysis of diameter changes. The Dmax/Dmin ratio ranged between 1.02 and 1.10, with limited variability. The maximum value was observed at 500 mL/min and 8 kPa (Figure 8), while under the remaining experimental conditions, the conduit exhibited stable behavior, with values close to unity, reflecting uniform and limited deformation.

### 3.4. Results of Mechanical Tests

The results of tensile tests are presented separately for both SIS conduits and DPP-2; DPP-1 was excluded.

#### 3.4.1. Ring Tensile Test on SIS-1 Conduit

**Figure A3:**
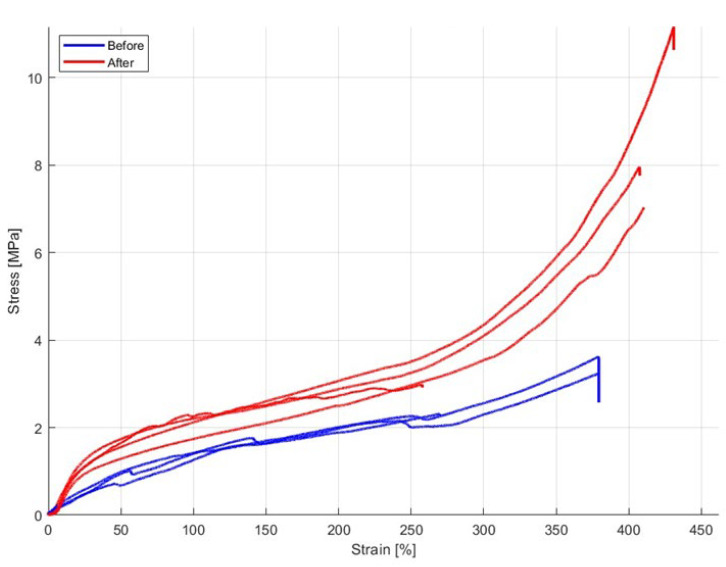
Stress–strain curves comparing SIS-1 samples in their native state (blue) and after SBF conditioning (red).

Samples from the SIS-1 conduit were subjected to ring tests before and after exposure to SBF. The resulting stress–strain curves are shown in Figure A3, while Table 1 summarizes the elastic moduli (E1 and E2), UTS, and FS values before and after SBF exposure. After exposure, an approximately 3.6-fold increase in modulus E1 and a 2.6-fold increase in modulus E2 were observed, accompanied by higher FS (approximately 10%) and UTS (2.4-fold) values, indicating material strengthening following functional testing.

#### 3.4.2. Ring Tensile Test on SIS-2 Conduit

**Figure A4:**
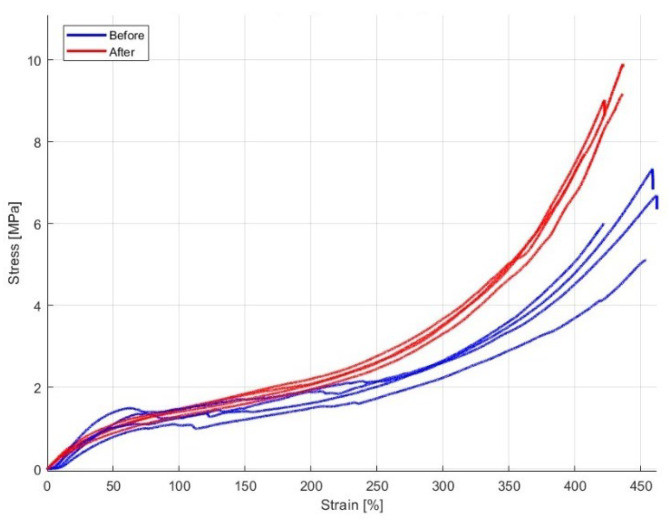
Stress–strain curves comparing SIS-2 samples in their native state (blue) and after DW conditioning (red).

Samples from the SIS-2 conduit were analyzed before and after exposure to distilled water. The corresponding stress–strain curves are shown in Figure A4, while the mechanical parameters are summarized in Table 2. An increase in stiffness was observed in both the initial and final regions of the curves, indicating post-test mechanical strengthening (1.6-fold for E1 and 1.75-fold for E2). FS values remained approximately unchanged (decrease of approximately 5%), whereas UTS showed an increase compared to pre-test values.

#### 3.4.3. Tensile Test on DPP-2 Conduit

**Figure A5:**
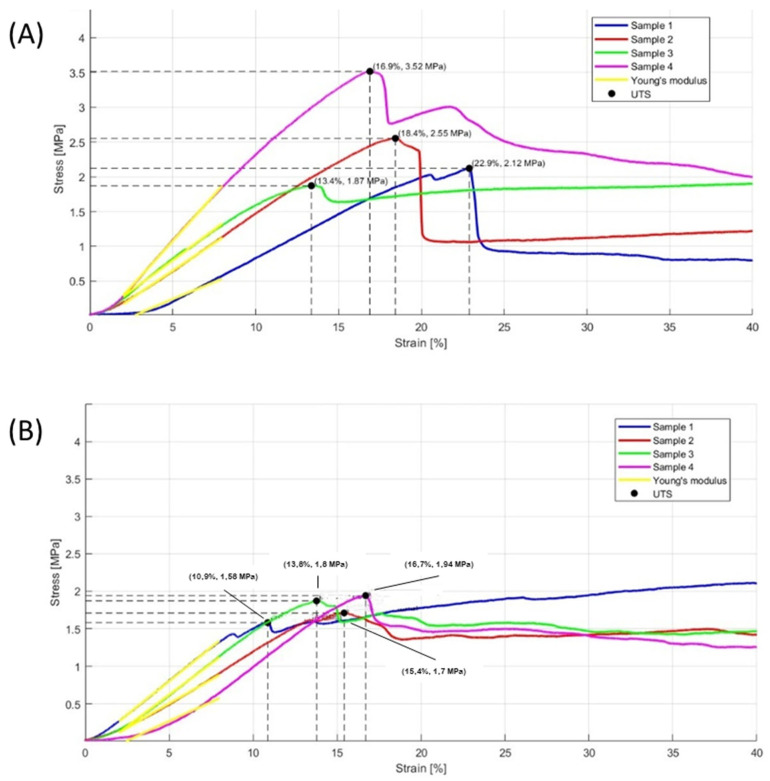
Stress–strain curves of DPP-2 samples before (blue) and after (red) exposure to distilled water (DW).

Regarding the decellularized pericardium, only samples from the DPP-2 conduit were biomechanically investigated. The stress–strain curves before and after exposure to SBF are shown in Figure A5. The black points marking UTS values indicate the onset of tissue tearing and define the upper limit of the region of interest. Beyond this point, the curves exhibit discontinuities and oscillations due to the progressive rupture of the sutures; nevertheless, the conduit retains residual load-bearing capacity over wide strain ranges, sometimes exceeding 600%. For this reason, only the initial modulus (E1) was calculated. Mechanical parameters (E1, UTS, and FS) are reported in Table 3. The data show no increase in stiffness after testing, while FS values decreased and UTS values remained unchanged or slightly decreased compared to pre-test values.

## 4. Discussion

Regarding the image processing methods, the U-Net++ model with a ResNet-50 encoder achieved excellent performance on both the training and test sets, with Dice, IoU, Precision, Recall, and F1-score values all exceeding 0.998. Quantitative analysis revealed highly compact metric distributions, indicating minimal variability across images and, consequently, strong generalization capability of the model. Qualitative evaluation of the acquired images confirmed the spatial accuracy of segmentation, with predicted masks well aligned with the ground truth even in low-contrast areas or in the presence of minor artifacts. However, despite the excellent overall performance, some limitations can be identified. The model was trained and validated on images acquired under specific lighting and contrast conditions; therefore, extreme variations could reduce segmentation accuracy. Pixel-level error maps showed that errors are concentrated along the conduit edges, where minor annotation uncertainties may affect the precise measurement of diameters. Moreover, the quality of the ground truth masks directly influences the model’s performance, and annotation errors could introduce bias during training. Finally, while training on 3300 images proved sufficient for the experimental conditions analyzed, the application of the model to new conduits with different optical characteristics may require additional adaptation strategies or retraining.

The model demonstrated high accuracy and robustness in segmenting the investigated conduits, providing a reliable basis for the automated extraction of quantitative parameters. The identified limitations mainly concern the limited number of samples, variability at the conduit edges, and generalization to significantly different imaging conditions. These aspects should be addressed in future studies or when applying the model beyond the current experimental context.

The functional tests enabled a detailed characterization of both SIS and DPP conduits tested with different fluids (SBF and distilled water), under varying flow rates and pressures. In general, the hybrid conduits maintained stable lumen profiles, with Dmax/Dmin ratio values close to unity within the physiological range of 370–500 mL/min and 8–11 kPa, suggesting that these conduits may be considered suitable candidates for applications requiring controlled and physiologically compatible deformations. In particular, SIS-1 tested in SBF exhibited minimal deformation, with Dmax/Dmin values between 1.01 and 1.02 (Figure 5). SIS-2 tested in distilled water showed greater variability, particularly at high flow rates and pressures, with peaks up to 2.14 (Figure 6); however, behavior remained stable (1.00–1.04) within the physiological range. DPP-1 tested in distilled water showed moderate dilation, with values between 1.02 and 1.16 under physiological conditions and higher peaks (up to 1.48) under more demanding conditions (Figure 7). DPP-2 tested in SBF maintained nearly uniform deformations (1.02–1.10) (Figure 8).

The biomechanical assessment enabled the characterization of the hybrid conduits’ response to load, highlighting variations induced by exposure to different fluids and by the application of cyclic stresses during previous functional tests. For the SIS-1 conduit tested in SBF, the comparison between pre- and post-test samples showed an increase in mechanical strength, with higher E1 (+259%), E2 (+162%), UTS (+138%), and FS (+10%) values. These results suggest a possible mechanical conditioning effect induced by the hydrodynamic test, which may promote better fiber alignment within the biological tissue matrix, thereby enhancing the material’s ability to withstand flow-induced mechanical stresses. Similarly, SIS-2 tested in distilled water showed increased UTS (+42%), E1 (+64%), and E2 (+75%) values, while FS remained essentially unchanged (−5%). Overall, following functional tests with both SBF and distilled water, SIS conduits exhibited mechanical modifications: their response to load became more regular, suggesting a conditioning effect likely attributable to prolonged exposure to pulsatile fluid.

DPP samples exhibited greater irregularities and higher variability compared to SIS conduits. In particular, DPP-1 tested in distilled water was not subjected to mechanical testing, as the presence of sutures prevented reliable measurements. DPP-2 tested in SBF showed decreased E1 (−17%), FS (−21%), and UTS (−29%) values, while still displaying overall consistent mechanical behavior among the investigated samples.

The preliminary functional evaluation presented in this work is essential for assessing the response of hybrid conduits under simulated physiological conditions. Beyond their mechanical characterization, the biological behavior of these materials has been encouraging. In particular, conduits made of decellularized porcine pericardium demonstrated good biocompatibility both in vitro and in vivo, with no signs of calcification or significant inflammatory response after 8 weeks of implantation, suggesting that the hybrid configuration supports tissue integration while limiting adverse reactions [18]. These findings are consistent with previous reports indicating that decellularized pericardium can effectively promote host cell infiltration and neovascularization without pathological mineralization. Similarly, small intestinal submucosa (SIS) has shown excellent in vitro biocompatibility, supporting cell adhesion, proliferation, and metabolic activity when tested with relevant cell lines, in line with previous evidence highlighting its ability to promote endothelial and smooth muscle cell growth [19]. These results further confirm that both biological components of the hybrid conduits are suitable for biomedical applications requiring close interaction with host tissues.

It is important to note that the present pilot study included the first in-house fabricated hybrid conduits and, being based on only four samples, yielded limited results. However, considering that the conduits were prepared manually, they demonstrated remarkable resilience to pressures well above the tested range without bursting (approximately 20–25 kPa), as assessed after the experiments. Nevertheless, repeated manipulation of the conduits significantly affected their structural integrity. Therefore, future experiments are planned involving a larger number of samples prepared according to a standardized fabrication protocol. The intended tests will employ a pulse generator allowing for modification of input signals (Vivitro pump), and will comprehensively characterize the mechanical properties of the developed constructs, including compliance, fatigue strength, diameter–pressure–flow relationships, maximum pressure limits, and associated safety factors accounting for the maximum pressures that may occur in the potentially replaced organs.

## 5. Conclusions

This work investigated the functional and mechanical performance of hybrid conduits derived from small intestinal submucosa (SIS) and decellularized porcine pericardium (DPP), tested in simulated body fluid and distilled water. Supported by advanced image analysis methods, the study demonstrated that hybrid conduits represent promising alternatives to synthetic prostheses for cardiovascular and urological applications.

A major contribution of this work lies in the integration of classical image processing with deep learning-based segmentation. While traditional methods provided robust results for conduits with clear contours, the U-Net++ model with a ResNet-50 encoder enabled accurate lumen tracking in more challenging cases, confirming the value of artificial intelligence in biomedical image analysis. Segmentation errors remained marginal and were primarily localized at the conduit edges, suggesting that improvements in the imaging setup—such as multi-angle acquisition and reference baselines for resting diameters—would further strengthen quantitative evaluations.

Functional characterization showed that SIS conduits, particularly SIS-1 tested in SBF, maintained highly stable lumen profiles within the physiological range, underscoring their suitability for vascular and urological graft applications requiring controlled compliance. DPP conduits, although affected by structural inhomogeneity due to sutures, also displayed acceptable performance, with DPP-2 tested in SBF exhibiting relatively consistent deformation patterns.

Mechanical testing revealed that SIS conduits exhibited increased stiffness following hydrodynamic conditioning, which may enhance long-term durability. In contrast, DPP samples showed more variable responses: as discussed above, the presence of sutures limited both test feasibility and mechanical uniformity.

Several limitations of the current approach must be acknowledged, as they restrict the generalizability of the conclusions. First, structural variability in the conduits, particularly in DPP, constrained the reproducibility of both mechanical and functional results. Second, the single-perspective imaging setup limited the ability to fully characterize three-dimensional deformations. Most importantly, due to the limited sample size, the current findings lack sufficient statistical power to support definitive conclusions and must therefore be interpreted as strictly preliminary. Finally, it was not possible to assess the effects of fluid exposure (SBF vs. distilled water) on the potential calcification of hybrid conduits: SEM analysis proved inadequate for detecting calcium deposits on the inner surface of the conduits.

Future work should focus on refining the experimental setup, including enhanced pressure control, multi-angle imaging, and baseline calibration of conduit diameters. Broader experimental campaigns on larger sample sets will be essential to improve statistical robustness. Additionally, microscopic analyses—such as scanning electron microscopy (SEM)—could provide valuable insights into tissue-level modifications, including fiber rearrangement induced by hydrodynamic stresses. The development of scalable and reproducible manufacturing processes, reducing reliance on manual suturing, will be critical to minimize variability and support clinical translation. However, before any clinical application, a thorough biological and functional assessment—both in vitro and in animal models—will be mandatory to evaluate biocompatibility, long-term mechanical performance, and in vivo tissue response under physiologically relevant conditions.

## Figures and Tables

**Table 1:** Values of initial elastic modulus (E1), final elastic modulus (E2), ultimate tensile strength (UTS), and failure strain (FS%) for SIS-1 conduit samples before and after exposure to SBF.

Sample	UTS [MPa]	FS [%]	E1 [MPa]	E2 [MPa]
Before exposure to SBF				
1	2.312	270	1.46	0.67 *
2	3.621	379	2.49	1.54
3	3.235	379	1.94	1.28
Mean ± SD	3.06 ± 0.67	342 ± 63.2	1.96 ± 0.52	1.16 ± 0.45
After exposure to SBF				
1	2.985	258	6.83	0.80 *
2	11.163	431	7.45	4.59
3	7.033	410	5.75	3.25
4	7.959	407	8.09	3.53
Mean ± SD	7.29 ± 3.37	376 ± 80.0	7.03 ± 0.99	3.04 ± 1.6

* Values calculated in the 240–257% strain range due to early failure.

**Table 2:** Values of initial elastic modulus (E1), final elastic modulus (E2), ultimate tensile strength (UTS), and failure strain (FS%) for SIS-2 conduit samples before and after exposure to DW.

Sample	UTS [MPa]	FS [%]	E1 [MPa]	E2 [MPa]
Before exposure to DW				
1	5.110	454	0.56	1.51
2	6.686	462	1.12	2.03
3	7.340	459	2.67	2.26
4	6.006	422	2.86	2.69
Mean ± SD	6.29 ± 0.95	449 ± 18.4	1.80 ± 1.14	2.12 ± 0.49
After exposure to DW				
1	9.027	422	2.92	4.03
2	7.705	407	3.42	3.18
3	9.904	436	2.92	4.29
4	9.170	436	2.52	3.33
Mean ± SD	8.95 ± 0.92	426 ± 13.8	2.95 ± 0.37	3.7 ± 0.53

**Table 3:** Values of elastic modulus (E1), ultimate tensile strength (UTS), and failure strain (FS%) for DPP-2 samples before and after exposure to SBF.

Sample	UTS [MPa]	FS [%]	E1 [MPa]
Before exposure to SBF			
1	2.12	22.90	10.01
2	2.55	18.44	15.95
3	1.87	13.37	17.53
4	3.52	16.90	26.40
Mean ± SD	2.52 ± 0.72	17.90 ± 3.95	17.47 ± 0.78
After exposure to SBF			
1	1.58	10.86	17.83
2	1.71	15.42	12.79
3	1.87	13.77	16.67
4	1.94	16.70	10.25
Mean ± SD	1.78 ± 0.16	14.19 ± 2.52	14.39 ± 3.5

## Data Availability

All data supporting the findings of this study are presented within the paper.

## Supplementary Materials

The following supporting information can be downloaded at: https://www.mdpi.com/article/10.3390/polym18111283/s1, Video S1.

## Abbreviations

The following abbreviations are used in this manuscript:

CF AR	Chronoflex AR
DMAc	N,N-dimethylacetamide
DPP	Decellularized porcine pericardium
DW	Distilled water
HDs	Hybrid conduits
PBS	Phosphate-Buffered Saline
SBF	Simulated body fluid
SIS	Small intestinal submucosa

## Appendix A

### Appendix A.1

**Table A1:** Detailed SBF composition.

Component	Amount
NaCl	8.035 g
NaHCO_3_	42 mL of 0.1 M solution
KCl	0.225 g
K_2_HPO_4_·3H_2_O	0.165 g
MgCl_2_·6H_2_O	0.146 g
CaCl_2_	0.292 g
Na_2_SO_4_	0.072 g
TRIS (Tris(hydroxymethyl)aminomethane)	6.118 g
1 M HCl	Up to approximately 60 mL, for pH adjustment up to approximately 60 mL, for pH adjustment (pH = 7.40 ± 0.02 at 36.5 °C)

### Appendix A.2

Temporal synchronization was achieved by identifying the peaks of maximum pressure and maximum conduit deformation. This approach enabled localization of the conduit section exhibiting the greatest deviation from the mean diameter, thereby highlighting the most pronounced oscillations—dilations and contractions—over time. Once the pressure peaks and diameter variations were identified, the data were cyclically aligned by shifting the diameter arrays and corresponding image filenames so that the frame of maximum diameter coincided with the frame of maximum pressure. Since the pressure signal was sampled at different time intervals than the imaging frames used to compute the diameters, it was subsequently linearly interpolated onto the same temporal grid. Such a simplification was necessary since it is not possible to measure the development of the pressure waveform along the flexible conduit. The pressure wires could be theoretically inserted into the conduit to measure the temporary pressure in the investigated cross-section but such catheters would affect the local flow conditions and increase uncertainties. This allowed each frame to be simultaneously associated with the conduit image, the measured diameter, and the interpolated pressure value. The resulting dynamic visualization—compiled into a final video—displayed, for each frame, the conduit image alongside the instantaneous diameter and pressure, enabling both qualitative and quantitative analysis of conduit deformation (Video S1). Finally, from the synchronized dataset, the maximum, minimum, and mean diameters—as well as the Dmax/Dmin ratio—were computed, yielding quantitative parameters for the functional characterization of conduit mechanical behavior.
